# Association between Maternal Non-Coding Interferon-λ Polymorphisms and Congenital Zika Syndrome in a Cohort from Brazilian Northeast

**DOI:** 10.3390/v13112253

**Published:** 2021-11-10

**Authors:** Átila Duque Rossi, Fabio Rueda Faucz, Adriana Melo, Girlene Souza de Azevedo, Paula Pezzuto, Ohanna Cavalcanti de Lima Bezerra, Fernanda Saloum de Neves Manta, Tamiris Azamor, Bruno Luiz Fonseca Schamber-Reis, Amilcar Tanuri, Milton Ozório Moraes, Renato Santana Aguiar, Constantine A. Stratakis, Cynthia Chester Cardoso

**Affiliations:** 1 Laboratório de Virologia Molecular, Instituto de Biologia, Universidade Federal do Rio de Janeiro, Rio de Janeiro 21941-902, Brazil; rossi.ad@icloud.com (Á.D.R.); paulapezzuto81@gmail.com (P.P.); atanuri1@gmail.com (A.T.); santanarnt@gmail.com (R.S.A.); 2 Section on Endocrinology and Genetics, Eunice Kennedy Shriver National Institute of Child Health and Human Development, National Institutes of Health, Bethesda, MD 20814, USA; fabio.faucz@nih.gov (F.R.F.); stratakc@cc1.nichd.nih.gov (C.A.S.); 3 Instituto de Pesquisa Professor Joaquim Amorim Neto (IPESQ), Campina Grande 58406-115, Brazil; asomelo@gmail.com (A.M.); girlene.giz@gmail.com (G.S.d.A.); 4 Laboratório de Hanseníase, Instituto Oswaldo Cruz Fiocruz, Rio de Janeiro 21040-900, Brazil; ohannacavalcanti@gmail.com (O.C.d.L.B.); femanta@yahoo.com.br (F.S.d.N.M.); tamifiocruz@yahoo.com.br (T.A.); milton.moraes@fiocruz.br (M.O.M.); 5 Faculdade de Ciências Médicas de Campina Grande, Núcleo de Genética Médica, Centro Universitário UniFacisa, Campina Grande 58408-326, Brazil; bruno.reis@fcm.edu.br; 6 Laboratório de Biologia Integrativa, Departamento de Genética, Ecologia e Evolução, Instituto de Ciências Biológicas, Universidade Federal de Minas Gerais, Belo Horizonte 31270-901, Brazil

**Keywords:** Zika, type III interferon, polymorphism, congenital Zika syndrome, genetic susceptibility

## Abstract

Congenital Zika syndrome (CZS) is characterized by a diverse group of congenital malformations induced by ZIKV infection during pregnancy. Type III interferons have been associated with placental immunity against ZIKV and restriction of vertical transmission in mice, and non-coding single-nucleotide polymorphisms (SNPs) on these genes are well known to influence susceptibility to other viral infections. However, their effect on ZIKV pathogenesis has not yet been explored. To investigate whether maternal non-coding SNPs at *IFNL* genes are associated with CZS, 52 women infected with ZIKV during pregnancy were enrolled in a case–control association study. A total of 28 women were classified as cases and 24 as controls based on the presence or absence of CZS in their infants, and seven Interferon-λ non-coding SNPs (rs12980275, rs8099917, rs4803217, rs4803219, rs8119886, rs368234815, rs12979860) were genotyped. The results of logistic regression analyses show an association between the G allele at rs8099917 and increased susceptibility to CZS under a log-additive model (_adjusted_OR = 2.80; _95%_CI = 1.14–6.91; *p* = 0.02), after adjustment for trimester of infection and genetic ancestry. These results provide evidence of an association between Interferon-λ SNPs and CZS, suggesting rs8099917 as a promising candidate for further studies on larger cohorts.

## 1. Introduction

Zika fever is an arthropod-borne viral disease transmitted mostly by *Aedes* mosquitoes’ bites, although alternative routes such as transfusion and vertical transmission have also been described [1,2]. Infection by Zika virus (ZIKV) is, in general, self-limiting and asymptomatic, but mild symptoms such as fever, myalgia, arthralgia, rash and conjunctivitis may be present [2]. ZIKV-infected individuals may also develop an autoimmune outcome: Guillain–Barré syndrome, characterized by subacute monophasic ascending flaccid paralysis [3]. However, most of the ZIKV burden relates to severe outcomes observed after vertical transmission events, known as congenital Zika syndrome (CZS). CZS harbors different neurological congenital malformations that may include microcephaly, ocular anomalies, arthrogryposis, lissencephaly, hydrocephaly, brain calcifications and postnatal neurodevelopmental impairment [2]. About 4 to 8% of children born from women infected with ZIKV exhibit malformations, suggesting that additional risk factors are required for the CZS outcome [4]. Preliminary results of a comparative twin study have suggested that CZS may be influenced by genetic and/or epigenetic variations [5]. We have previously shown the association between maternal variations at genes encoding adenylate cyclases and susceptibility to CZS, reinforcing the relevance of genetic factors to ZIKV pathogenesis [6]. Later, a role for variations at the *TNF* and *TLR3* genes in susceptibility to CZS was also suggested [7].

The placenta is considered a frontline defense against vertical transmission of infectious agents to the fetus. Interferons (IFN) play a major role in this scenario by regulating the innate immune response at the maternal–fetal interface [8]. The mechanisms by which ZIKV trespasses the placental barrier and inflicts fetal damage remain to be elucidated. However, its ability to inhibit the type I interferon pathway has already been described, with recent evidence suggesting Interferon-λs as critical innate immune factors to protect the placenta environment from ZIKV infection [9].

Non-coding single-nucleotide polymorphisms (SNPs) in IFN-λ genes have been widely investigated in the context of viral infections, and the SNPs rs368234815, rs12979860 and rs8099917 were consistently associated with spontaneous virus clearance and therapy response in HCV-positive individuals. Additional variations such as rs12980275, rs8109886, rs4903217 and rs4803219 were also associated with hepatitis C outcomes in distinct populations [10,11]. Moreover, SNP rs12979860 was also associated with the severity of HSV infection, while rs368234815 and rs8099917 were, respectively, associated with human cytomegalovirus (HCMV) retinitis and reactivation [12,13]. Here, we conducted a case–control association study to assess whether these *IFNL* non-coding polymorphisms are associated with CZS in a cohort of pregnant women from the Brazilian Northeast region.

## 2. Materials and Methods

The present study enrolled 52 women infected with ZIKV during pregnancy, who attended to Instituto de Pesquisa Professor Joaquim Amorim Neto (IPESQ), in the city of Campina Grande, Brazilian Northeast region, from 2015 to 2017. Subjects were classified as cases (*n* = 28) based on the presence of neurological congenital malformations such as microcephaly, brain calcifications, ventriculomegaly, cerebellar hypoplasia, arthrogryposis, lissencephaly and/or hydrocephaly due to ZIKV infection in infants (congenital Zika syndrome, CZS). The control group (*n* = 24) consisted of infected women who gave birth to healthy children. Clinical diagnosis was performed by specialized physicians from IPESQ, and all samples were also submitted to molecular/serological tests to confirm ZIKV infection and exclude other arboviral infections (dengue and chikungunya) or infections associated with congenital malformations (STORCH). The criteria for clinical and laboratory diagnosis were described in detail in a previous study [6]. The present study was approved by the institutional review board, and written informed consent was obtained from all participants. General characteristics of cases and controls are described in Table S1.

Genomic DNA was extracted from frozen blood samples using QIAamp DNA Blood Mini Kit following the manufacturer’s recommendations (QIAGEN^®^). Seven non-exonic SNPs located on Interferon-λ genes or intergenic regions were selected based on their previous described associations with viral infections. SNPs rs12980275, rs4803219, rs8109886 and rs8099917 are in intergenic regions, while rs4803217 and rs368234815 are in regulatory regions of *IFNL3* and *IFNL4*, respectively. The SNP rs12979860 is intronic (*IFNL4*) (Figure S1). Genotyping was performed by PCR amplification followed by Sanger sequencing. Sequence analysis and genotype calling were performed using Geneious software (Biomatters, New Zealand). For genetic ancestry profiling, 46 informative indels were amplified in a single multiplex PCR and analyzed using capillary electrophoresis [14]. Genotypes were determined after analysis using GeneMapper software (Thermo Fisher Scientific, Waltham, MA, USA). Proportions of European, Native American, and African ancestries were estimated using the software STRUCTURE as described [14].

Statistical analyses were performed using R software (version 3.4) with the package “SNPassoc”. Deviations from Hardy–Weinberg equilibrium (HWE) were assessed by χ^2^ tests. Linkage disequilibrium (LD) patterns were determined using the r2 statistic. The association between each SNP and the development of CZS was estimated by unconditional logistic regression models adjusted for gestational age at the time of infection (1st trimester or later) and genetic ancestry estimates (as continuous variables). An LD heatmap and an SNP *p*-value plot were generated with the package “snp.plot”.

## 3. Results

To investigate whether maternal Interferon-λ genes are associated with CZS, we conducted a case–control association study considering 7 SNPs in 52 women from the Northeast region of Brazil. All SNPs were detected in our cohort, with minor allele frequencies ranging from 19% (rs8099917) to 46% (rs4803217) (Figure S1). Deviation from HWE was only observed for SNP rs368234815, which was then removed from analysis. Genetic ancestry profiling showed predominance of European ancestry (57% and 59% for controls and cases, respectively) in this population, followed by African and Native American patterns, which were equally represented (21%) among controls (Table S1). Although we did not observe a statistical difference in infection during the first trimester between cases and controls in our cohort (*p* = 0.30; chi-squared test with one degree of freedom), there was a clear tendency of higher incidence in the case group, consistent with previous literature findings [15].

**Figure 1:**
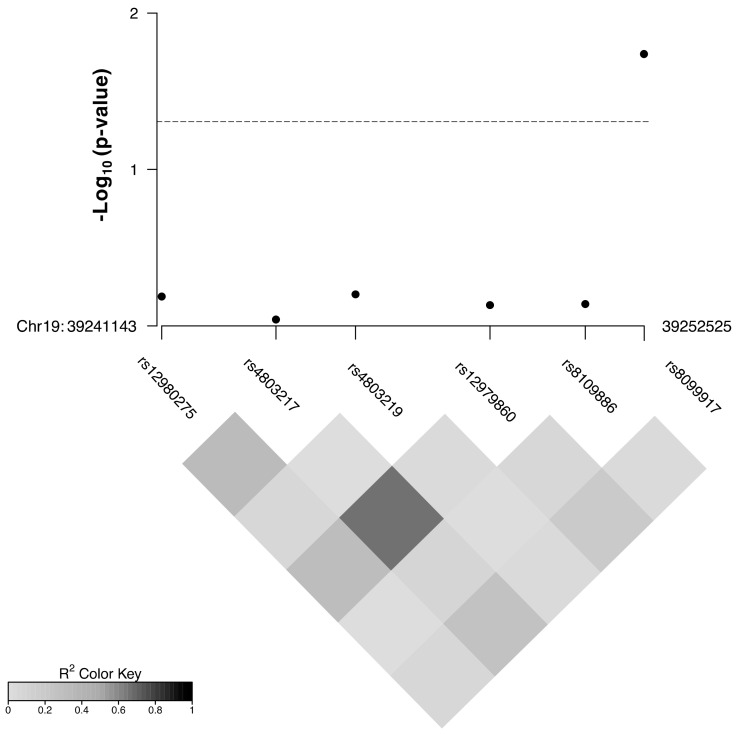
Linkage disequilibrium patterns and association between non-coding polymorphisms in Interferon-λ genes and congenital Zika syndrome in Campina Grande cohort. The *p*-values were obtained from log-additive logistic regression models adjusted for genetic ancestry and trimester of infection during pregnancy (first trimester or other). Dashed line stands for *p*-value = 0.05.

The results of linkage disequilibrium analyses (Figure 1) showed strong LD only between rs12979860 and rs4803217 (r^2^ = 0.79). Therefore, SNP rs4803217 was excluded from further analysis. The remaining SNPs were analyzed by different logistic regression models considering genetic ancestry and trimester of infection as adjustment variables due to their biological relevance. The results obtained show an association between maternal rs8099917 (G) and increased risk of CZS under the log-additive model either before (OR = 2.41; _95%_CI = 1.02–5.71; *p* = 0.03) or after adjustment for covariates (_adjusted_OR = 2.80; _95%_CI = 1.14–6.91; *p* = 0.02) (Figure 1; Table 1). The remaining SNPs were not associated with CZS in our cohort (Figure 1; Table S2).

## 4. Discussion

The influence of Interferon-λ non-coding SNPs on HCV spontaneous and treatment-induced clearance is among the strongest associations found to date in the context of infectious disease, which has made them valuable clinical tools for monitoring HCV prognosis [11]. IFN-λ shares many common signaling elements with type I interferons but acts in a more specific manner. IFN-λ induces interferon-stimulated genes (ISGs) preferentially in cells of epithelial origin, such as hepatocytes, and affects endothelial permeability in the blood–brain barrier [16]. Due to its involvement in the innate immune response at key sites of flaviviral pathogenesis, IFN-λ’s impact on ZIKV infection has been explored in animal models, and a role at the maternal–fetal interface has already been described [9]. However, the impact of Interferon-λ SNPs on CZS remains poorly understood. We have previously investigated a role for coding variants at Interferon-λ genes in CZS in the same cohort, but no significant association was found [6]. Here, after non-coding Interferon-λ polymorphism analysis, we could identify an association between the maternal G allele at SNP rs8099917 and increased risk of CZS. This is the same variation that has been consistently associated with the outcomes of HCV infection, including spontaneous clearance and drug response [10]. Moreover, the genotype rs8099917 GG is considered a risk genotype to antiviral therapy non-responsiveness, while the rs8099917 TT genotype favors spontaneous clearance of HCV infection, suggesting a functional role of this SNP during viral replication/pathogenesis [17]. In the present study, OR estimates suggested a risk effect for the maternal rs8099917 GG genotype on CZS in codominant and recessive models. Previous studies have suggested that the G allele is associated with reduced IFNL3 expression, suggesting that the risk effect observed might result from an impaired antiviral response that could favor ZIKV vertical transmission [10]. Although our results of codominant and recessive models for this SNP could not reach statistical significance (probably due to the small sample size that led to underrepresentation of GG individuals), we found an effect for the G allele under log-additive models. In fact, the rs8099917 G allele was the least frequent (19%) among the SNPs tested in this study.

Genetic association studies with small sample sizes are highly vulnerable to random allelic fluctuation. Nevertheless, the frequency obtained for rs8099917 G in our cohort was close to the 17% expected for European populations from the 1000Genomes database, consistent with the predominant European ancestry from our cohort (Figure S1). Despite this fact, the association observed cannot be extrapolated to other populations, since CZS is a complex phenotype and depends on the interaction of varying genetic and environmental risk factors.

Genetic variation in rs368234815 also plays an important role in HCV prognosis, since carriers of the ΔG allele produce IFN-λ 4, another IFN-λ family member, whose expression is negatively associated with HCV spontaneous clearance [11]. We were unable to test the association between rs368234815 and CZS due to its deviation from HWE. This may be a consequence of the small sample size but could also suggest an impact of this polymorphism on susceptibility to ZIKV infection per se, since all subjects enrolled in this study were ZIKV positive.

Taken together, our data provide evidence of a role for maternal genetics at Interferon-λ genes in the CZS outcome. Further analyses with larger sample sizes are strongly encouraged to validate our findings and enable exploring possible haplotype effects.

## Figures and Tables

**Table 1:** Association between polymorphism rs8099917 and congenital Zika syndrome in a cohort from Campina Grande.

rs8099917 Genotype	Controls (%)	Cases (%)	OR (95% CI)	OR (95% CI) *
T/T	16 (66.7)	12 (42.9)	Reference	Reference
T/G	7 (29.2)	10 (35.7)	1.90 (0.56–6.46)	2.04 (0.57–7.38)
G/G	1 (4.2)	6 (21.4)	8.00 (0.85–75.86)	10.01 (1.0–100.47)
	24	28	*p* = 0.09	*p* = 0.06
T/T	16 (66.7)	12 (42.9)	Reference	Reference
T/G—G/G	8 (33.3)	16 (57.1)	2.67 (0.86–8.27)	2.98 (0.90–9.88)
	24	28	*p* = 0.08	*p* = 0.07
T/T—T/G	23 (95.8)	22 (78.6)	Reference	Reference
G/G	1 (4.2)	6 (21.4)	6.27 (0.7–56.4)	7.47 (0.80–70.24)
	24	28	*p* = 0.05	*p* = 0.06
log-additive (G)	24	28	**2.41 (1.02–5.71)**	**2.80 (1.14–6.91)**
			***p* = 0.03**	***p* = 0.02**

* Results of logistic regression models adjusted for American and African genetic ancestry (continuous) and the trimester of infection (first trimester or later) during pregnancy. Significant associations are highlighted in **bold**.

## Data Availability

All the data collected during this study may be provided upon request.

## Supplementary Materials

The following are available online at https://www.mdpi.com/article/10.3390/v13112253/s1, Figure S1: Genome position and frequency of each Interferon-λ SNP in 1000Genomes populations and Campina Grande cohort; Table S1: Characteristics of the cohort from Campina Grande, Brazil; Table S2: Association analysis between other non-coding polymorphisms at Interferon-λ cluster and congenital Zika syndrome in the cohort from Campina Grande, Brazil.
