# Supplementary material for: Association between Maternal Non-Coding Interferon-λ Polymorphisms and Congenital Zika Syndrome in a Cohort from Brazilian Northeast
Source: Viruses. 2021 Nov 10;13(11):2253. doi: 10.3390/v13112253 (PMC8622836; doi:10.3390/v13112253)
Supplement: Supplementary file 1 [file viruses-13-02253-s001.zip › viruses-1424850-si proofdone.pdf]

Supplementary Tables:

Table S1. Characteristics of the cohort from Campina Grande, Brazil.

	**Controls (N = 24)**	**Cases (N = 28)**
**Gestational Age***		
First trimester	14 (58.33)	20 (71.4)
After	10 (41.67)	8 (28.6)
**Genetic Ancestry (%)**		
European	57.3 ± 14.30	59.7 ± 13.80
African	21.0 ± 10.80	21.0 ± 11.84
American	21.7 ± 9.30	19.3 ± 8.55
**ZIKV Diagnosis**		
PCR+	13 (54.16)	9 (32.14)
IgM+	8 (33.33)	0 (0)
IgG+	3 (12.5)	19 (67.86)
**Child's Outcome**		
Microcephaly**	-	22 (78.6)
Other	-	6 (21.4)

Results are represented as N (%) for categorial variables while proportions of each genetic ancestry are represented as mean ± standard deviation for each group. *Gestational age when ZIKV related symptoms occurred or when ZIKV infection was confirmed by PCR or serology. **Individuals in this category may present other malformations in addition to microcephaly such as brain calcifications, ventriculomegaly, cerebellar hypoplasia, arthrogryposis, lissencephaly and/or hydrocephaly.

Table S2. Association analysis between other non-coding polymorphisms at *Interferon-λ* cluster and Congenital Zika Syndrome in the cohort from Campina Grande, Brazil

Genotype	Controls (%)	Cases (%)	OR (95% CI)	OR (95% CI)*
**rs12980275**				
A/A	13 (54.2)	19 (67.9)	Reference	Reference
G/A	8 (33.3)	6 (21.4)	0.51 (0.14 - 1.83)	0.64 (0.16 -2.56)
G/G	3 (12.5)	3 (10.7)	0.68 (0.12 - 3.93)	0.80 (0.13 - 4.98)
			p= 0.57	p= 0.82
log-additive	24	28	0.72 (0.33 – 1.60)	0.82 (0.36 – 1.90)
			p= 0.42	p= 0.65
**rs4803219**				
C/C	9 (37.5)	14 (50)	Reference	Reference
C/T	15 (62.5)	14 (50)	1.13 (0.38 – 3.38)	1.33 (0.42 - 4.20)
T/T	0	0	-	-
			p= 0.83	p= 0.63
log-additive	24	28	1.13 (0.38 – 3.38)	1.33 (0.42 - 4.20)
			p= 0.83	p= 0.63
**rs8109886**				
A/A	13 (54.2)	15 (53.6)	Reference	Reference
A/C	11 (45.8)	11 (39.3)	0.87 (0.28 -2.65)	0.87 (0.27 – 2.79)
C/C	0	2 (7.1)	-	-
			p= 0.63	p= 0.37
log-additive	24	28	1.27 (0.49 – 3.32)	1.20 (0.43 – 3.36)
			p= 0.63	p= 0.73
**rs12979860**				
C/C	7 (29.2)	10 (35.7)	Reference	Reference
T/C	13 (54.2)	12 (46.4)	0.70 (0.20 - 2.41)	0.72 (0.20 - 2.56)
T/T	4 (16.7)	5 (17.9)	0.88 (0.17 - 4.47)	0.81 (0.14 - 4.74)
			p= 0.85	p= 0.88
log-additive	24	28	0.89 (0.41 – 1.97)	0.86 (0.37 – 2.02)
			p= 0.78	p= 0.74

*Results of logistic regression models adjusted for Native American and African genetic ancestry (continuous) and the trimester of infection (first trimester or later) during pregnancy.

Supplementary Figure S1:

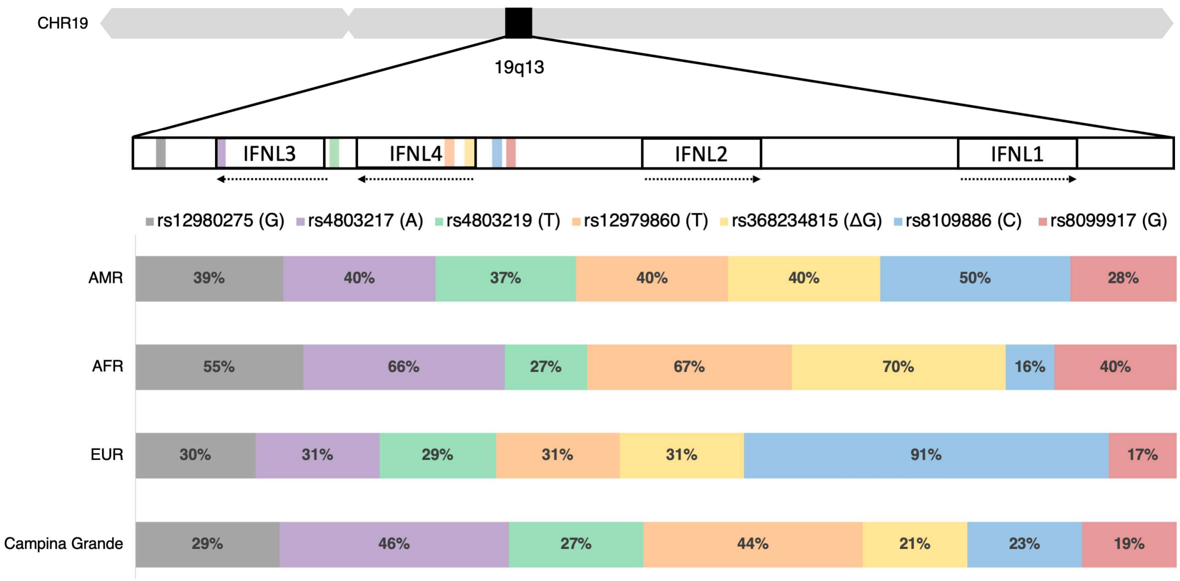

Figure S1. Genome position and frequency of each Interferon-λ SNP in 1000Genomes populations and Campina Grande cohort. SNPs rs12980275, rs4803219, rs8109886 and rs8099917 are in intergenic regions while rs12979860 is intronic. The SNP rs4803217 is in the 3'UTR region of *IFNL3* and rs368234815 is positioned in the promoter region of *IFNL4*. rs368234815 G allele creates an open reading frame that allows *IFNL4* expression. AMR = Native Americans; AFR= Africans; EUR = Europeans.
